# Selectively improving nikkomycin Z production by blocking the imidazolone biosynthetic pathway of nikkomycin X and uracil feeding in *Streptomyces ansochromogenes*

**DOI:** 10.1186/1475-2859-8-61

**Published:** 2009-11-23

**Authors:** Guojian Liao, Jine Li, Lei Li, Haihua Yang, Yuqing Tian, Huarong Tan

**Affiliations:** 1 State Key Laboratory of Microbial Resources, Institute of Microbiology, Chinese Academy of Sciences, Beijing 100101, PR China; 2 Graduate School of Chinese Academy of Sciences, Beijing 100039, PR China

## Abstract

**Background:**

Nikkomycins are a group of peptidyl nucleoside antibiotics and act as potent inhibitors of chitin synthases in fungi and insects. Nikkomycin X and Z are the main components produced by *Streptomyces ansochromogenes*. Of them, nikkomycin Z is a promising antifungal agent with clinical significance. Since highly structural similarities between nikkomycin Z and X, separation of nikkomycin Z from the culture medium of *S. ansochromogenes *is difficult. Thus, generating a nikkomycin Z selectively producing strain is vital to scale up the nikkomycin Z yields for clinical trials.

**Results:**

A nikkomycin Z producing strain (sanPDM) was constructed by blocking the imidazolone biosynthetic pathway of nikkomycin X via genetic manipulation and yielded 300 mg/L nikkomycin Z and abolished the nikkomycin X production. To further increase the yield of nikkomycin Z, the effects of different precursors on its production were investigated. Precursors of nucleoside moiety (uracil or uridine) had a stimulatory effect on nikkomycin Z production while precursors of peptidyl moiety (L-lysine and L-glutamate) had no effect. sanPDM produced the maximum yields of nikkomycin Z (800 mg/L) in the presence of uracil at the concentration of 2 g/L and it was approximately 2.6-fold higher than that of the parent strain.

**Conclusion:**

A high nikkomycin Z selectively producing was obtained by genetic manipulation combined with precursors feeding. The strategy presented here might be applicable in other bacteria to selectively produce targeted antibiotics.

## Background

**Figure 1:**
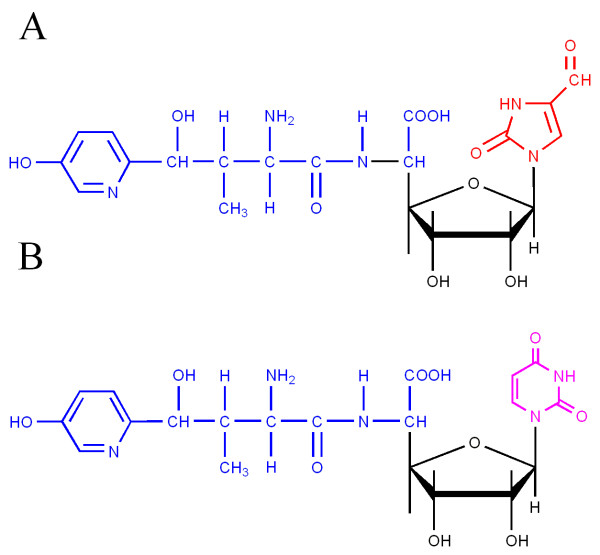
**Chemical structures of nikkomycin X (A) and Z (B), the main components produced by *Streptomyces ansochromogenes *TH322**.

Nikkomycins, a group of peptidyl nucleoside antibiotics produced by *Streptomyces ansochromogenes *[1] and *Streptomyces tendae *[2], are potent competitive inhibitors of chitin synthase. These antibiotics are structurally similar to UDP-*N*-acetylglucosamine which is the natural substrate of chitin synthase. So they can inhibit the growth of insects, acarids, yeasts, and filamentous fungi [3]. Nikkomycin X and Z, main components produced by both *S. ansochromogenes *and *S. tendae*, are the most active structures (Fig. 1). They are composed of hydoxypyridylhomethreonine (nikkomycin D) and a 5-aminohexuronic acid *N*-glucosidically bound to uracil in nikkomycin Z or to 4-formyl-4-imidazolin-2-one (imidazolone) in nikkomycin X. The corresponding nucleoside moieties are designated as nikkomycin Cz and Cx. Nikkomycin I and J, produced as minor components by *S. tendae *but not by *S. ansochromogenes*, are structurally analogous to nikkomycin X and nikkomycin Z and contain glutamic acid peptidically bound to the 6'-carboxyl group of aminohexuronic acid [4]. In the past few years, particular attention has been drawn to nikkomycin Z for its significant activity against the highly chitinous, pathogenic, dimorphic fungi *Coccidioides immitis *and *Blastomyces dermatitidis *and phase I/II clinical research of nikkomycin Z as an orphan product for treatment of occiciodomycosis is undergoing [5]. Meanwhile, nikkomycin Z has synergetic effect with azoles and echinocandins against *Candida albicas *and *Aspergillus fumigatus *[6-8]

**Figure 2:**
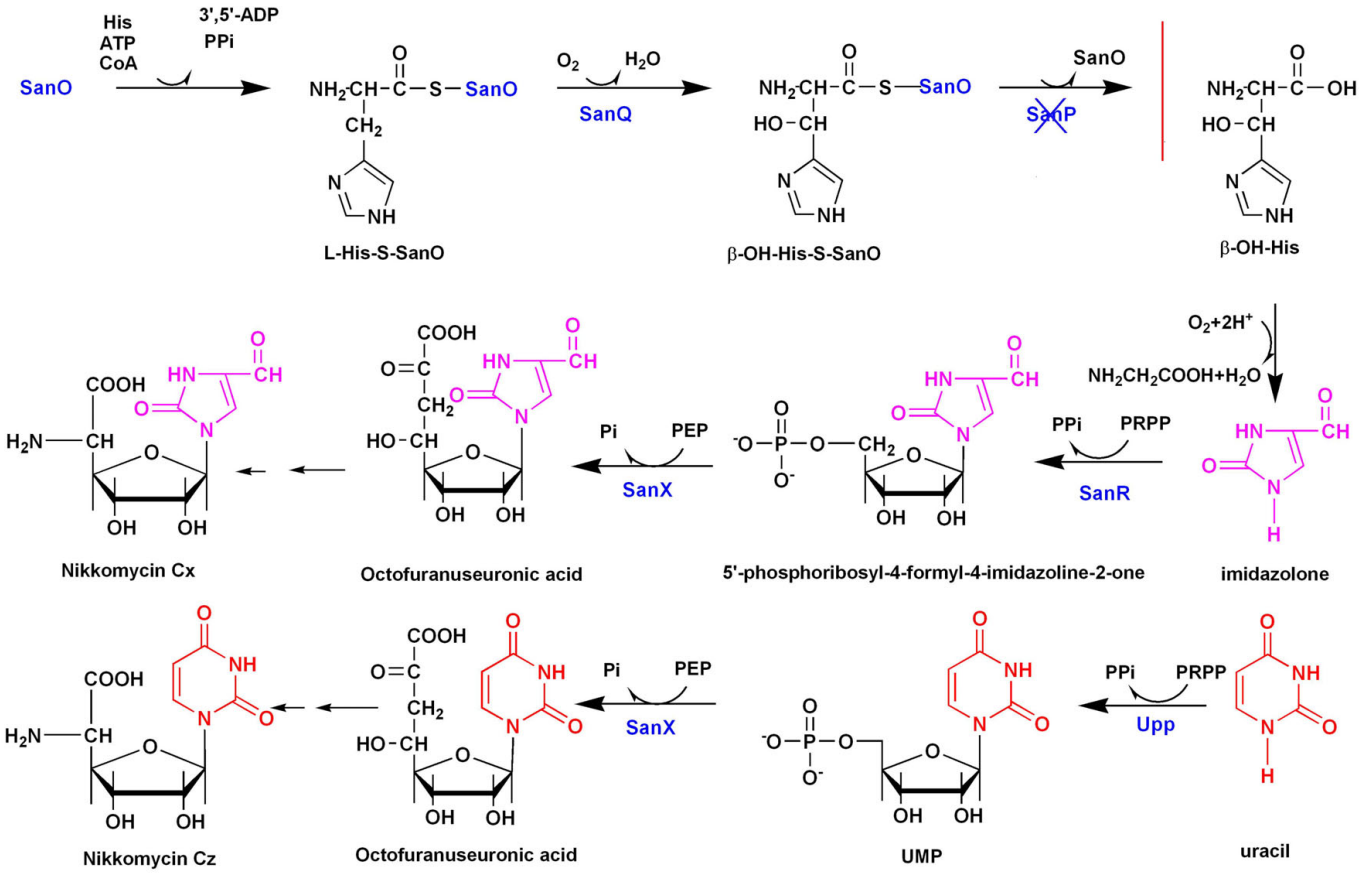
**Biosythetic pathway of nucleoside moiety of nikkomycin in *S. ansochromogenes***. Some biosynthetic steps were omitted. SanO, L-His-specific didomain NRPS; SanP, type II thioesterase; SanQ, heme hdyroxylase; SanR, uracil phosphoribosyltransferase; SanS, enolpyruvyl transferase; Upp, uracilmphosphoribosyltransferase; PRPP, 5-phosphoribosyl-alpha-1-diphosphate; PEP, phosphoenolpyruvate;. Disruption of *sanP *resulted in blocking the synthesis of β-OH-His.

Separation of nikkomycin Z from the culture medium is difficult due to the highly structural similarity among nikkomycins. This is much more complicated by its iosmer nikkomycin X. Thus, the abolishment of nikkomycin X, I and J production is crucial for scaling up nikkomycin Z yields for clinical trials. Studies such as strain improvement, optimization of the production medium and fermentation process, have significantly increased the yield of nikkomycins, but strains selectively producing nikkomycin Z remain unavailable [9]. Recently, considerable progresses have been made in understanding nikkomycin biosynthesis in *S. ansochromogenes *and *S. tendae*. Nikkomycin biosynthetic cluster has been identified in both strains and subsequent biochemical characterizations have elucidated the functions of some genes. Among them, *sanO*, *sanQ, sanR *and *SanX *were involved in biosynthesis of nikkomycin Cx and Cz (Fig. 2) [10-12]. Gene disruption of *sanO *or *sanQ *resulted in the blocking of nikkomycin X biosynthesis in *S. ansochromogenes *7100, but had no effect on the production of nikkomycin Z. These studies raised the possibility that the blocking of nikkomycin X biosynthesis by genetic manipulation in *S. ansochromogenes *might generate a dedicated nikkomycin Z producing strain, since nikkomycin I and J were not produced by *S. ansochromogenes*.

In this paper, a strain which produced high-level of nikkomyicns obtained by traditional strain improvement was chosen as the parent strain for genetic manipulation. A nikkomycin Z selectively producing strain was generated by blocking the imidazolone biosynthetic pathway of nikkomycin X. The subsequent uracil feeding further enhanced the yield of nikkomycin Z.

## Results

### Construction of the nikkomycin Z selectively producing strain

**Figure 3:**
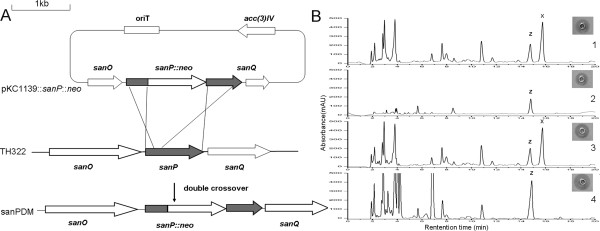
**Insertional inactivation of *sanP *and analysis of nikkomycin production**. (A) Diagram of *sanP *gene disruption in *S. ansochromogenes *TH322. A kanamycin antibiotic resistance cassette was inserted into the *Bam*HI site of *sanP*. (B) Bioassay and HPLC analysis of nikkomycin production. X, nikkomycin X; Z, nikkomycin Z; 1, culture filtrates from TH322; 2, culture filtrates from sanPDM; 3, culture filtrates from the complementary strain; 4, culture filtrates from sanPDM feeding with 2 g/L uracil. The strains were inoculated in liquid SP medium for 144 h.

To obtain an ideal strain only producing nikkomycin Z, the disruption of *sanP *in *S. ansochromogenes *TH322 was performed (Fig. 3A). *sanP *is an homologue of *nikP2 *of *S. tendae *(sharing 95% identity), which is vital for the biosynthesis of imidazolone that is an unique part of nikkomycin X [13]. The resulting *sanP *disruption mutant (sanPDM) was passed through five generations in the absence of antibiotic pressure. The progeny still conferred kanamycin resistance, indicating the resulting sanPDM was genetically stable.

**Figure 4:**
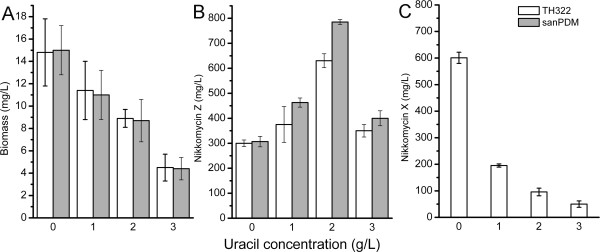
**Effect of uracil on biomass and nikkomycin production**. (A) Effect of uracil on the biomass of *S. ansochromogenes *TH322 and sanPDM; (B) Effect of uracil on the nikkomycin Z production in *S. ansochromogenes *TH322 and sanPDM; (C) Effect of uracil on the nikkomycin X production in *S. ansochromogenes *TH322.

*S. ansochromogenes *sanPDM produced almost the same amounts of nikkomycin Z (300 mg/L) as TH322 (Fig. 3B), while no nikkomycin X was produced by sanPDM. Biomass of sanPDM was approximately equal to that of TH322 (Fig. 4A).

In *S. ansochromogenes*, *sanP *is located in one of the operons [1]. To exclude the potential polar effect of *sanP *disruption on the loss of nikkomycin X, the complementary plasmid pIM229::*sanP *constructed with wild type *sanP *under the control of the *erm*E* promoter was introduced into the sanPDM. The introduction of pIM229::*sanP *into sanPDM restored nikkomycin X production. HPLC analysis and bioassay showed that the amounts of nikkomycins produced by the complemented strain were almost the same as those produced by the parent strain (Fig. 3B). The disruption and complementation experiments suggested that *sanP *was a key determinant in nikkomycin X biosynthesis and disruption of *sanP *did not affect the production of nikkomycin Z.

### Effect of precursor of nucleoside moiety on nikkomycin Z production

To further increase the yield of nikkomycin Z, the effects of different precursors on its production were investigated. Recent studies revealed that UMP was the direct precursor for the biosynthesis of the nucleoside moiety of nikkomycin Z [14] and UMP could be synthesized from uracil or uridine through salvage pathway. So, the effect of supplementation of varying concentration (1 g/L-3 g/L) of uracil on nikkomycin production was measured in *S. ansochromogenes *TH322 and sanPDM. As shown in Fig. 4B, uracil had a stimulatory effect on nikkomycin Z production in both strains and uracil supplementation led to much more nikkomycin Z production in sanPDM than that in TH322 at all concentrations tested in the study. Addition of uracil (1 g/L to 2 g/L) resulted in increased nikkomycin Z production from 450 mg/L to 800 mg/L in sanPDM or increased from 375 mg/L to 630 mg/L in TH322, whereas nikkomycin X production was decreased from 195 mg/L to 96 mg/L in TH322 (Fig. 4C). The change of biomass was observed in both strains. Addition of uracil (2 g/L) in the culture medium resulted in reduced the biomass by approximate 40% compared to that of the control (Fig. 4A). Addition of more uracil (3 g/L) resulted in significant inhibition of cell growth and reduced the final yield of nikkomycins in both strains. These results demonstrated that addition higher concentration of uracil might inhibit cell growth.

To determine uracil consumption, uracil residue in the culture medium after fermentation was measured. As shown in Table 1, uracil could be hardly detected when uracil was fed at the concentration of 1.5 g/L, while 0. 484 g/L uracil left when 2 g/L uracil was fed. These results indicated that only 1.5 g/L uracil can be utilized during the fermentation. In the subsequent experiments, a saturated concentration of uracil (2 g/L) was used to achieve the maximum production of nikkomycin Z.

**Table 1:** The uracil residue, nikkomycin Z production and biomass in culture medium of sanPDM with uracil supplementation

Uracil (g/L)	Nikkomycin Z production (mg/L)	Biomass (g/L)	Uracil residue (g/L)
0	300 ± 20	14.8 ± 2.2	ND
0.5	400 ± 15	13.4 ± 1.7	ND
1.0	480 ± 18	11.0 ± 2.2	ND
1.5	790 ± 23	9.5 ± 1.4	0.080 ± 0.020
2.0	800 ± 18	9.1 ± 1.0	0.484 ± 0.031

ND, not detected in culture medium

Similar studies were carried out to determine the effect of uridine supplementation on nikkomycin Z production. Addition of uridine (0.5 g/L to 2 g/L) resulted in enhancing nikkomycin Z production from 320 mg/L to 400 mg/L without detectable effect on the biomass (data not shown). Thus, uridine exerted a less stimulatory effect than uracil on nikkomycin Z production.

### Effect of precursor of peptidyl moiety on nikkomycin Z production

Precursor feeding and biochemical studies have shown that L-glutamate and L-lysine are precursors for biosynthesis of peptidyl moiety of nikkomycin Z [15,16].

L-lysine and L-glutamate (0.5, 1 and 2 g/L) were added separately to the culture medium fed with 2 g/L uracil in order to study their influences on the nikkomycin Z production in *S. ansochromogenes *sanPDM. It was observed that L-lysine (0.5 g/L to 1 g/L) had no remarkable effect on the nikkomycin Z production and the biomass (Table 2). Further increasing the concentration of lysine resulted in decreased the yield of nikkomycin Z and inhibited the cell growth. Similar results were obtained when L-glutamate was added into the culture medium. As both L-lysine and L-glutamate are needed to synthesize the peptidyl moiety, the effect of supplementing L-lysine and L-glutamate in culture medium on the yield of nikkomycin Z was studied (Table 2). It was observed that there was no increase in nikkomycin Z production with L-lysine and L-glutamate feeding compared to that without supplementation. These results together demonstrated that L-lysine and L-glutamate might not be the limiting-factor of nikkomycin Z production in the complex SP medium. So, the optimum condition to obtain maximum productivity of nikkomycin Z (800 mg/L) was culturing the sanPDM in the presence of uracil at the concentration of 2 g/L

**Table 2:** Effect of L-lysine and L-glutamate on nikkomycin Z production in sanPDM in the presence of 2 g/L uracil

Culture conditions	Nikkomycin Z production (mg/L)	Biomass (g/L)
Control	785 ± 10	9.9 ± 1.9
L-lysine		
0.5%	780 ± 16	10.2 ± 2.1
1%	700 ± 40	9.2 ± 1.3
2%	500 ± 9	5.3 ± 1.9
L-glutamate		
0.5%	770 ± 30	9.7 ± 1.7
1%	720 ± 12	9.5 ± 2.1
2%	400 ± 23	4.8 ± 1.6
L-lysine+L-glutamate		
0.5%+0.5%	750 ± 30	9.6 ± 1.5
1%+1%	740 ± 24	9.8 ± 2.0

## Discussion

Nikkomycin Z is a potential antifungal agent with clinical significance. However, the presence of its isomer, nikkomycin X during fermentation interferes with the purification of nikkomycin Z and increases the production cost. Thus, we chose a nikkomycin high producing strain (*S. ansochromogenes *TH322) to perform genetic manipulation and obtained a nikkomycin Z selectively producing strain (sanPDM). *sanP *encoded a type II thioesterase which hydrolytically released SanO from β-OH-His-SanO to form β-OH-His (Fig. 2). Disruption of it resulted in blocking the synthesis of β-OH-His and failed production of nikkomycin Cx and nikkomycin X, whereas, disruption of *sanP *did not affect nikkomycin Z production because uracil, the base in nikkomycin Z, was biosynthesized via *de novo *or salvage pathway. These results were mainly in accordance with those studies in *S. tendae *[13]. Disruption of *nikP2 *almost abolished the nikkomycin X production, while disruption of *sanP *in *S. ansochromogenes *resulted in eliminating nikkomycin X production. Loss of the nikkomycin X production in the fermentation would significantly improve the recovery of nikkomycin Z during large scale manufacturing process.

Feeding precursors have been proved to be a successful strategy to increase the yield of targeted antibiotics, such as feeding ornithine to enhance clavulanic acid production in *S. clavuligerus *[17]. We found that addition of uracil (2 g/L) increased production of nikkomycin Z by 2-fold while suppressed nikkomycin X production by 85% in TH322. A possible explanation to these results was that UMP derived form uracil competed with imidazolone for incorporation into core of nikkomycin. A similar study on salinosporamide B in *Salinispora tropica *was verified, finding that addition of butyric acid-the precursor of salinosporamide B to the culture medium resulted in increased the production of salinosporamide B by approximately 3.2-fold while inhibited the production of salinosporamide A by 26% [18].

Uracil supplementation led to considerable increase of nikkomycin Z production, while its high concentration (more than 2 g/L) significantly inhibited cell growth and reduced the yield of nikkomycin Z. Our results were in accordance with the reports of Krishna [19]. Uracil (2 g/L) could improve the rifamycin SV production by 505 mg/L in *Amycolatopsis mediterranei *MV35R, while higher concentration inhibited both the cell growth and rifamycin SV production. The reason why higher concentration of uracil sharply inhibited the cell growth was still unclear. Uridine had a slight effect on nikkomycin Z production. Uracil salvage pathway had been identified in *S. grieus *and *S. coelicolor *[20] and genes involving in uracil transport (uracil permase) and transformation to UMP (uracil phosphoribosyltransferase) were highly conserved in *Streptomyces*. However, gene encoding uridine kinase that converted uridine into UMP had not been found in *Streptomyces *so far. So, we speculated that the stimulatory effect of uridine on nikkomycin Z production may be due to the partial degradation of uridine to uracil and uracil acted as precursor to participate in nikkomycin Z biosynthesis.

## Conclusion

Combining genetic manipulation with uracil feeding enabled us to selectively enhance the yield of nikkomycin Z. As nikkomycin Z could be recovered without additional separation from other related components in sanPDM, it would simplify the downstream purification process and lower the production cost.

## Methods

### Strains, plasmids and culture conditions

*Streptomyces ansochromogenes *TH322, a high-level producer of nikkomycins (300 mg/L nikkomycin Z and 600 mg/L nikkomycin X), was collected in our laboratory. *S. ansochromogenes *sanPDM, the nikkomycin Z selectively producing strain created in this study was deposited with the China General Microbiological Culture Collection Center (CGMCC) and assigned the accession number was CGMCC-3086. *Candida albicans *CGMCC No 2.2086 was used as indicator strain for nikkomycin bioassays. *Escherichia coli *JM109 was used as host for cloning and subcloning. *E. coli *ET12567 containing pUZ8002 was used for conjugal transfer according to the established protocol [21]. *Streptomyces-E. coli *shuttle plasmid pKC1139 for gene disruption was provided by Prof. Keith Chater (John Innes Center, Norwich, UK)

*S. ansochromogenes *TH322 was grown on mannitol/soya (MS) medium at 28°C [21] for 6 days to form spores. For genomic DNA isolation, the *S. ansochromogenes *TH322 and *sanP *disruption mutant (sanPDM) were grown in YEME medium for 2 days. SP medium (3% mannitol, 1% soluble starch, 0.5% soy peptone and 0.8% yeast extract, pH 6.0) was used for nikkomycin production.

When necessary, antibiotics were used at the following concentrations: apramycin, 10 μg ml^-1 ^in YEME or MS for *S. ansochromogenes *TH322, 100 μg ml^-1 ^in LB for *E. coli*; kanamycin, 10 μg ml^-1 ^in YEME or MS for *S. ansochromogenes *TH322, 100 μg ml^-1 ^in LB for *E. coli*.

### Primers and PCR conditions

The oligonucletide primers used to amplify the *sanP *were P1 (5'-GTGGCACCGCGACAGGCCG-3') and P2 (5'-TCAACTCTCATCGGCTC-3'). P3 (5'-GCGGCCAGCTACTTCCGGGAC-3') and P4 (5'-GCAGAAAGGCCGAGCGCATGT-3') were used to check the *sanP *disruption mutant. The PCR was performed using KOD (Toyobo, Japan): an initial denaturation at 95°C for 4 min followed by 30 cycles of amplification (95°C for 1 min, 62°C for 30 sec, and 68°C for 1 min) and additional 10 min at 68°C.

### Construction of *sanP *disruption mutants

Disruption of *sanP *was performed by gene replacement via homologous recombination. For this purpose, a 2.5 kb *Pst*I-*Sac*I DNA fragment containing the complete *sanP *sequence was inserted into the *Pst*I-*Sac*I site of pBluescript II KS (-) to generate pBS::*sanP*. The kanamycin-resistance gene (*neo*) was isolated from pUC119::*neo *after digestion with *Bam*HI and *Kpn*I, blunted and inserted into the blunted *Bam*HI site of *sanP *in pBS::*sanP *to generate pBS::*sanP*::*neo*. A 3.5 kb *Pst*I-*Sac*I fragment from pBS::*sanP*::*neo *was inserted into the *Pst*I-*Sac*I site of pIJ2925 to give pIJ2925::*sanP*::*neo*. The resulting plasmid was then digested with *Bgl*II and the resulting 3.5 kb fragment was inserted into the *Bam*HI site of pKC1139 to generate pKC1139::*sanP*::*neo*.

The resulting plasmid pKC1139::*sanP*::*neo *was passed through *E. coli *ET12567/pUZ8002 and then introduced into *S. ansochromogenes *TH322 by conjugal transfer according to the established techniques. The resulting transformants were inoculated on MS plates to form spores. Gray spores were harvested and spread on MM plates containing kanamycin as the resistance selection. After incubating at 42°C for 3 days, the colonies that conferred kanamycin resistance (Kan^R^) and apramycin sensitivity (Apr^S^) were selected and further confirmed as *sanP *disruption mutatants by PCR using the sanPDM genomic DNA as template.

### Complementation of the *sanP *mutant

For complementation analysis, complete *sanP *DNA fragment was amplified from *S. ansochromogenes *TH322 genomic DNA by PCR using P1 and P2 primers. The amplified fragment was subsequently inserted into the *Eco*RV site of pIM229 under the control of promoter of *erm*E* [22]. The resulting recombinant plasmid pIM229::*sanP *was then integrated into the chromosomal *attB *site of *sanP *disruption mutant by conjugal transfer.

### Fermentation of *S. ansochromognes*

Spores of *S. anchromogenes *TH322 or sanPDM were inoculated in YEME. The cultures were grown at 28°C on a rotary shaker (220 rpm) for 48 h and used as seed cultures. 1 ml (0.5% V/V) of seed culture was inoculated into flasks containing 50 ml of SP medium, and then fermented at 28°C on a rotary shaker (200 r.p.m) for 6 days.

### Quantification of uracil in the culture filtrates

Uracil was analyzed according to the method of Gao [23] by HPLC using a Zorbax SB-C18 reverse-phase column at 254 nm with a 0.5 ml min^-1 ^flow rate at 25°C. The elution profile was a linear gradient of 0%-20% solution B (A = 5 mM ammonium acetate; B = methanol) over 15 min. An aliquot of 20 μL culture filtrates was injected for HPLC analysis.

### Bioassay and HPLC analysis of nikkomycins

The culture filtrates were harvested by centrifugation and the supernatant was filtered through a Minipore membrane (pore diameter 0.2 μm). Nikkomycin Z (Sigma, cat. No. N8028) was used as standard. HPLC analysis and bioassay of nikkomycins against *Candida albicans *were performed as described previously [24].

## Competing interests

The authors declare that they have no competing interests.

## Authors' contributions

GL carried out the experiments, wrote the draft manuscript and analyzed the primary data. JL, LL, HY and YT assisted with experimental design, data analysis. HT supervised the whole work and revised the manuscript. All authors read and approved the final manuscript.
